# Multiscale High-Level Feature Fusion for Histopathological Image Classification

**DOI:** 10.1155/2017/7521846

**Published:** 2017-12-31

**Authors:** ZhiFei Lai, HuiFang Deng

**Affiliations:** Department of Computer Science and Engineering, South China University of Technology, Guangzhou 510006, China

## Abstract

Histopathological image classification is one of the most important steps for disease diagnosis. We proposed a method for multiclass histopathological image classification based on deep convolutional neural network referred to as coding network. It can gain better representation for the histopathological image than only using coding network. The main process is that training a deep convolutional neural network is to extract high-level feature and fuse two convolutional layers' high-level feature as multiscale high-level feature. In order to gain better performance and high efficiency, we would employ sparse autoencoder (SAE) and principal components analysis (PCA) to reduce the dimensionality of multiscale high-level feature. We evaluate the proposed method on a real histopathological image dataset. Our results suggest that the proposed method is effective and outperforms the coding network.

## 1. Introduction

Medical image classification is one of the most important steps for disease diagnosis. In this paper, we would focus on the histopathological image classification task, which is a subset of medical image classification and can provide useful hints for doctor's disease diagnosis. Xu et al. [1] proposed a classification method based on a deep convolutional neural networks (DCNNs) to learn high-level feature to classify epithelial and stromal tissues. In their study, it is a binary classification task and it directly uses DCNN to complete the classification task rather than multiscale features. Cruz-Roa et al. [2] proposed a method to automate detection of of invasive ductal carcinoma in whole slide images of breast cancer with convolutional neural networks (CNNs). In [3], the authors used stacked sparse autoencoder (SSAE) to detect nuclei on breast cancer histopathological images where SSAE can learn discriminative high-level features. In [4], Esteva et al. proposed the method using DCNN to classify the skin cancer which can achieve dermatologist-level diagnosis and they evaluated the results on a dataset of 129,450 clinical images. It turns out that if the dataset is big enough, training the DCNN to deal with classification task usually works very well. Many current works [5–7] directly employ the DCNN to gain high-level feature classifying medical image but seldom incorporating the high-level feature solved this challenge problem; that is, their model just designed different convolutional layers and max-pooling layer and connected the soft-max layer in the end as classifier. However, it is pointed out that simply using DCNN to classify is not enough to gain a better performance and high efficiency especially in case of the limited histopathological image dataset. A convolutional autoencoder (CAE) based algorithm was proposed in [8], which encoded the image through CAE at first and then added the pre-extracted VGG features to a semisupervised CNN. Experimental results on a dataset of 2078 images indicated the proposed method can reduce the error rate of attribute and shape classification by 21.54% and 15.07%, respectively. Shu et al. [9] developed the Deep Transfer Networks (DTNs) to address the insufficient training examples challenge. They pretrained two SSAEs for text and image, respectively, and then continued to train SSAEs for shared representations called weakly shared DTNs. It proved the effectiveness of the model on NUS-WIDE dataset [10]. In the approach presented in [11], CNNs were accelerated with distributed GPUS via hybrid parallelism strategy called “Wheel” which transmitted most of the parameters in one server to reduce transmit data time. In addition, it fully run each GPU to reduce the idle time.

In order to improve the efficiency and accuracy of histopathological image classification, we propose a novel method based on the DCNN referred as coding network which extract two-layer high-level feature as multiscale high-level feature. This method is inspired by [12] that used DCNN to predict 10000 classes. But the difference is that our method fuses the high-level features to feed into another classifier and would put the multiscale feature into sparse autoencoder to reduce the dimensionality.

The remainder of this paper is organized as follows. We will describe the detailed procedure of extracting multiscale high-level feature and give an effective algorithm to reduce dimensionality of multiscale high-level feature in Section 2. In Section 3, we report our experiment in SDT dataset and the analysis of the model. Finally, we conclude the paper and give the future work in Section 4.

## 2. The Methodology of Proposed Model

### 2.1. Coding Network and Multiscale High-Level Feature Extraction

Our coding network contains six convolutional layers which follows by normalization layer called local response normalization reference by [13]. Before feeding into the softmax layer, it contains one full-connected layer and dropout [14] layer that includes setting to zero the output of each hidden neuron with probability 0.5. The normalization layer and dropout layer are important to coding network owing to improving the overall accuracy where it may get discriminative high-level feature. When training the coding network, the input is a fixed-size 140 × 140 × 3 RGB image.  Table 1 shows the detailed configure of coding network. The convolutional layer employs a filter with a receptive field of 7 × 7, 9 × 9, and 8 × 8 and a 1 pixel stride and 0 pixel padding. The pooling layer is performed over a 5 × 5 pixel window, with stride 2.

In extracting multiscale high-level feature, coding network is intent to extract high-level features. We extract the 6th convolutional layer and the full-connected layer combined as multiscale high-level features. The 6th convolutional layer is fixed to 2048 referred to as cfr, while the dimension of full-connected layer is 256 referred to as ffr. It directly fuses the cfr and ffr into one feature vector that can continue to feed into another softmax classifier to classify the histopathological image.

### 2.2. The Dimensionality Reduction of Multiscale High-Level Feature

We reduce the dimensionality of multiscale high-level feature vector based on the following considerations:

1. The extracted multiscale high-level feature which combines two-layer high-level features of coding network would lead to considerable computational complexity because of their high dimensions.
2. In addition, owing to the two different high-level features extracted from the coding network, it may inevitably bring about obvious correlation between them.

Therefore, we would use sparse autoencoder (SAE) to reduce multiscale high-level features because sparse autoencoder could gain more discriminative features due to cut the correlation between high-level features. We employ *xt* = {*x*^(1)^, *x*^(2)^, *x*^(3)^,…} representation of the extracted multiscale high-level feature. Sparse autoencoder is the three-layer neural network which is trying to train a target function $\hat{xt}\approx xt$, where $\hat{xt}$ is the output of sparse autoencoder and the second layer contains the sparse representation of original multiscale high-level $\hat{mh}$. It can be defined as follows:

(1) $$\begin{array}{c} \hat{mh}=f\left(\;W*xt+b\;\right). \end{array}$$

In (1), *f* is the activation function that we apply here *c*; *W* is the weight matrix and *b* is the bias. It is worth noting that the dimensionality of $\hat{mh}$ is lower than *xt*. In addition, sparse autoencoder minimized the cost function *J* as follows:

(2) $$\begin{array}{c} J=\frac{1}{m}\overset{m}{\underset{i=1}{\sum }}{\left(\;\hat{xt}-xt\;\right)}^{2}, \end{array}$$

where *m* is the total number of pathological images. However, in order to achieve the purpose of reducing dimensionality, we have to impose the sparse constraints overall cost function. Here, it gives the penalty term Kullback-Leibler (KL) divergence to cost function. It can be defined by

(3) $$\begin{array}{c} \overset{s_{2}}{\underset{j=1}{\sum }}KL\left(\;\rho ||\hat{\rho_{j}}\;\right), \end{array}$$

where *s*_2_ is the dimensionality of $\hat{mh}$; *ρ* a sparsity parameter that is closed to zero; $\hat{\rho_{j}}$ is the average activation of hidden unit *j* that can be defined as

(4) $$\begin{array}{c} \hat{\rho_{j}}=\frac{1}{m}\overset{m}{\underset{i=1}{\sum }}\left(\;\left(\;a_{j}^{\left(2\right)}\;\right)\left(\;x^{\left(i\right)}\;\right)\;\right), \end{array}$$

where *a*_*j*_^(2)^(*x*^(*i*)^) denotes the activation of sparse autoencoder hidden unit when the network is given a specific input *x*^(*i*)^. And $KL(\rho ||\hat{\rho_{j}})$ is given by

(5) $$\begin{array}{c} KL\left(\;\rho ||\hat{\rho_{j}}\;\right)=\rho \log\frac{\rho }{\hat{\rho_{j}}}+\left(\;1-\rho \;\right)\log\frac{1-\rho }{1-\hat{\rho_{j}}}. \end{array}$$

Therefore, the original cost function is substituted by

(6) $$\begin{array}{c} J_{sparse}=J+\beta \overset{s_{2}}{\underset{j=1}{\sum }}KL\left(\;\rho ||\hat{\rho }\;\right). \end{array}$$

*β* controls the weight of the sparsity penalty term. Minimizing the new cost function, we could gain the sparse representation of multiscale high-level features.

Principal components analysis (PCA) is a classical data dimensionality reduction algorithm in unsupervised feature learning area. A large amount of works [15–17] was based on PCA to solve the problem of high dimensionality, in which the feature will be decomposed as the linearly independent eigenvectors that choose the principal eigenvectors of the original feature. It is an effective method to reduce data dimensionality. Therefore, in order to demonstrate the effectiveness of SAE, it may use PCA algorithm for comparison to reduce the multiscale high-level feature dimensionality.

## 3. Experiment and Evaluation

### 3.1. Dataset Description and Experiment Setting

A real skin biopsy image dataset called SDT which contains 6 classes of skin disease images is used to evaluate the overall performance of the proposed method. The dataset is composed of 2019 images where each image is an RGB image of size 2048*∗*1536.  Table 2 summarizes the 6 classes in the dataset and employs T1, T2, T3, T4, T5, and T6 to label each category. In order to efficiently reduce the overfitting problem of the proposed method, we manually enlarge the SDT dataset through extracting random 960*∗*960 image patches from the original image. Then we resize the image patches to 140*∗*140. This not only saved the main information of medical image but also reduced the running time of our algorithm. The configuration of our experiment can be seen as follows. The dataset is divided into 3 parts: training set, validation set, and test set with a ratio 7 : 1 : 2. In addition, we trained our model on the extracted patches by 10-fold cross-validation. For the coding network, it had taken 45 epoches to get the network convergence. It trained the coding network using stochastic gradient descent with the batch size of 100 images, momentum of 0.9. Furthermore, the learning rate was initialized at 0.01, where it would adjust manually through training. The strategy is referenced by [13] which is to divide the learning rate by 10 when the validation error rate stopped improving.

### 3.2. Accuracy Analysis

In this section, it is necessary to compare the coding network with the multiscale high-level features for demonstrating the effectiveness and efficiency of proposed algorithm. Furthermore, multiscale feature + SAE (MSAE) will be compared with multiscale feature + PCA (MPCA) to verify the efficiency of dimensionality reduction. Quantitative evaluation is shown in Table 3 in which the overall accuracy reaches 86.2%, 92.6%, and 95.3%, respectively. From Table 3 it can be found that MSAE owns the best algorithm accuracy that outperforms the other two algorithms. In addition, it is obvious that MSAE and MPCA can gain perform better than coding network which demonstrates the efficiency of multiscale high-level features and the effectiveness of dimensionality reduction. All experiments are implemented in Matlab using MatConvNet package [18]. And all experiments are conducted on a computer with i5-6500 3.2 GHz CPU, 32 G main memory, and GTX1060 GPU.

In Table 4, it reports the experimental results which shows the comparison of algorithm accuracy in each category. We can see that no matter which algorithm is, it would gain better accuracy than overall performance in some cases. The unbalanced problem would account for this phenomenon. The more the samples of a category lie in the dataset, the better the accuracy can achieve. In addition, from Table 4 we clearly know that MSAE outperforms the coding network and MPCA in each category which confirms the effectiveness of our proposed method again.

**Figure 1:**
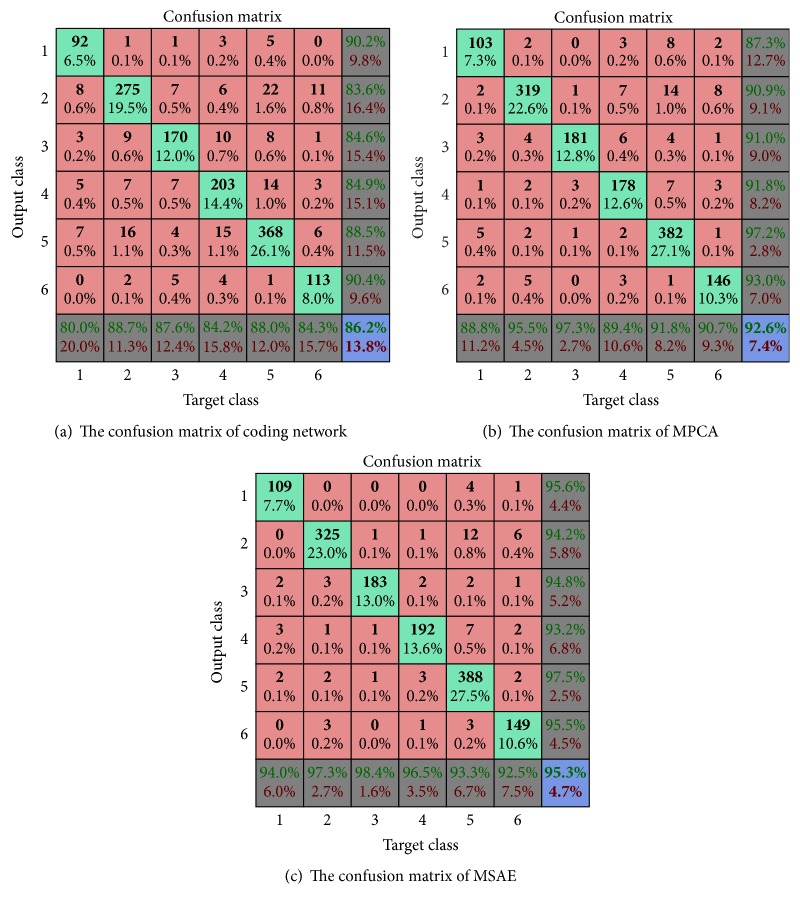
The confusion matrix of different algorithms.

**Figure 2:**
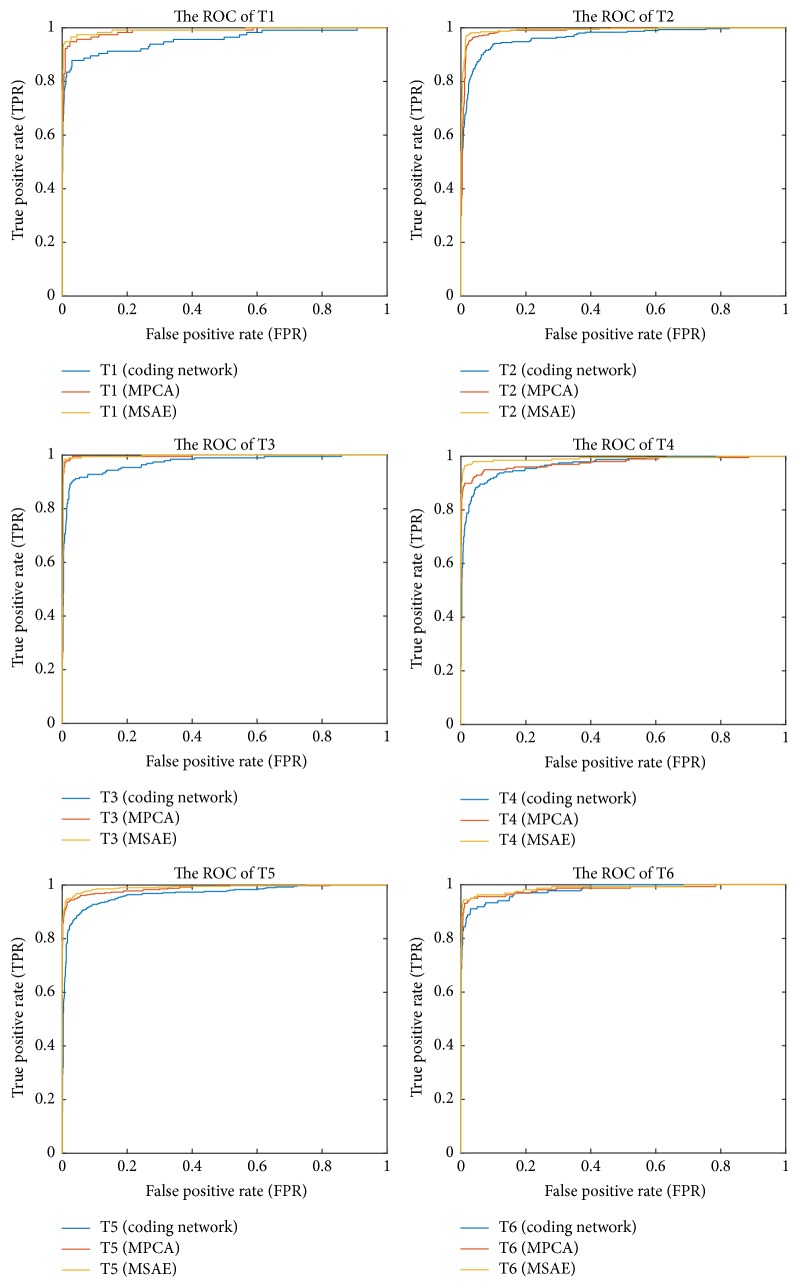
The ROC curve on different label of different algorithms.

Figure 1 shows the confusion matrix of different algorithms. In confusion matrix, the mint green cells represent the number and percentage of correct predictions made by the algorithm; the pink shaded cells exhibit the number and percentage of incorrect predictions. At the last row of confusion matrix, it gives the precision of each category, while it also gains the recall of each class in the last column of confusion matrix. The last diagonal element of confusion matrix represents the overall accuracy of algorithm. From Figure 1, MSAE can achieve better precision than MPCA and coding network in each class. The performance of our proposed model in minority class can clearly improve. In addition, it would draw a conclusion that T2 has the familiar structure with T5. Relative number samples of T2 that are predicted to T5 would account for this phenomenon. In order to better evaluate of the algorithms, we follow [19] using the receiver operating characteristic (ROC) of different classes as evaluation criteria.  Figure 2 compares the ROC curve of different classes. In addition, it computes the area under the curve (AUC) to more intuitive comparison.  Table 5 displays the mean AUC of different algorithms. It is obvious that the AUC of our proposed model can achieve 0.9912 which proves to be better than coding network and MPCA with 0.9617 and 0.9855, respectively.

## 4. Conclusion

We propose a multiclass histopathological image classification method which is based on the multiscale features. This method trains the coding network to extract high-level feature and combines one convolutional layer feature and full-connected feature as multiscale high-level feature. In order to solve the problem of high dimensionality and speed up the running time of the algorithm, we use SAE and PCA to reduce the dimensionality of multiscale feature. At last, we evaluate the method on the dataset SDT and the results show that MSAE outperforms PSAE and coding network. In our future work, we will train serval DCNNs to ensemble learning that can extract high-level features from these DCNNS. Specifically, we will design one deep learning framework referred to as multiscale high-level framework which gain the features. Meanwhile, fusing these high-level features feeds into the classifier to classify the histopathological images.

## Figures and Tables

**Table 1:** The configuration of the coding network.

Type	Kernel size/strid	Output size
Convolution	7 × 7 × 3/1	134 × 134 × 32
Convolution	7 × 7 × 32/1	128 × 128 × 32
Max pool	5 × 5/2	62 × 62 × 32
Convolution	9 × 9 × 32/1	54 × 54 × 64
Max pool	5 × 5/2	25 × 25 × 64
Convolution	7 × 7 × 64/1	19 × 19 × 64
Convolution	7 × 7 × 64/1	13 × 13 × 128
Max pool	6 × 6/2	4 × 4 × 128
Convolution	4 × 4 × 128/1	1 × 1 × 256
Full-connection	1 × 1 × 256/1	1 × 1 × 256
Softmax layer		1 × 1 × 6

**Table 2:** Six classes in SDT dataset with occurrence number.

Image category	Number of images	Label
Hyperpigmentation of basal cell layer	162	T1
Acanthosis	451	T2
Parakeratosis	265	T3
Hyperkeratosis	328	T4
Infiltration of lymphocytes	597	T5
Papillomatosis	216	T6

**Table 3:** The comparison of algorithm overall accuracy.

Algorithm	SDT dataset accuracy
Coding network	86.2%
MPCA	92.6%
MSAE	95.3%

**Table 4:** The comparison of algorithm accuracy in dataset SDT.

Label	Coding network	MPCA	MSAE
T1	80.0%	88.8%	94.0%
T2	88.7%	95.5%	97.3%
T3	87.6%	97.3%	98.4%
T4	84.2%	89.4%	96.5%
T5	88.0%	91.8%	93.3%
T6	84.3%	90.7%	92.5%

**Table 5:** The mean AUC of different algorithms.

Algorithm	The mean AUC of different algorithms
Coding network	0.9671
MPCA	0.9855
MSAE	0.9912
